# Effects of α-Synuclein Monomers Administration in the Gigantocellular Reticular Nucleus on Neurotransmission in Mouse Model

**DOI:** 10.1007/s11064-019-02732-5

**Published:** 2019-02-13

**Authors:** Ilona Joniec-Maciejak, Agnieszka Ciesielska, Łukasz A. Poniatowski, Adriana Wawer, Anna Sznejder-Pachołek, Ewa Wojnar, Piotr Maciejak, Dagmara Mirowska-Guzel

**Affiliations:** 1 Department of Experimental and Clinical Pharmacology, Centre for Preclinical Research and Technology (CePT), Medical University of Warsaw, Banacha 1B, 02-097 Warsaw, Poland; 2 Gladstone Institutes of Neurological Disease, 1650 Owens Street, San Francisco, 94158 USA; 3 Department of Neurosurgery, Maria Skłodowska-Curie Memorial Cancer Center, Institute of Oncology, W. K. Roentgena 5, 02-781 Warsaw, Poland; 4 Department of Neurochemistry, Institute of Psychiatry and Neurology, Sobieskiego 9, 02-957 Warsaw, Poland; 5 2nd Department of Neurology, Institute of Psychiatry and Neurology, Sobieskiego 9, 02-957 Warsaw, Poland

**Keywords:** α-Synuclein, Lewy bodies, Gigantocellular reticular nucleus, Neurotransmission, Parkinson’s disease

## Abstract

The aim of the study was to examine the Braak’s hypothesis to explain the spreading and distribution of the neuropathological changes observed in the course of Parkinson’s disease among ascending neuroanatomical regions. We investigated the neurotransmitter levels (monoamines and amino acid concentration) as well as tyrosine hydroxylase (TH) and transglutaminase-2 (TG2) mRNA expression in the mouse striata (ST) after intracerebral α-synuclein (ASN) administration into gigantocellular reticular nucleus (Gi). Male C57BL/10 Tar mice were used in this study. ASN was administrated by stereotactic injection into Gi area (4 μl; 1 μg/μl) and mice were decapitated after 1, 4 or 12 weeks post injection. The neurotransmitters concentration in ST were evaluated using HPLC detection. TH and TG2 mRNA expression were examined by Real-Time PCR method. At 4 and 12 weeks after ASN administration we observed decrease of DA concentration in ST relative to control groups and we found a significantly higher concentration one of the DA metabolites—DOPAC. At these time points, we also noticed the increase in DA turnover determined as DOPAC/DA ratio. Additionally, at 4 and 12 weeks after ASN injection we noted decreasing of TH mRNA expression. Our findings corresponds with the Braak’s theory about the presence of the first neuropathological changes within brainstem and then with time affecting higher neuroanatomical regions. These results obtained after administration of ASN monomers to the Gi area may be useful to explain the pathogenesis of Parkinson’s disease.

## Introduction

Parkinson’s disease (PD) is a one of the most common neurodegenerative disease affecting the central nervous system (CNS) characterized by the multitude of motor and non-motor clinical symptoms [1]. Considering the available long-term epidemiological data the age-standarized annual incidence of PD rates in high-income countries of 14 per 100.000 people while in elderly population (≥ 65 years) is estimated at 160 per 100.000 [2, 3]. The hallmark of PD motor manifestation include progressive tremor, rigidity, bradykinesia and postural instability [4]. The non-motor symptoms include, but are not limited to cognitive impairment, sleep disturbances, autonomic dysfunction and depression [5]. The major pathophysiological process occurring in the course of PD is associated with the degeneration of dopaminergic neurons in the substantia nigra (SN) and more specifically in its pars compacta (SNpc) within brainstem [6]. In this case, progressive loss of dopaminergic neurons results in reduction of the dopamine (DA) and its metabolites concentration in the striatum (ST), what directly leads to disruption of homeostasis between neurotransmitter systems and further disturbances of motor function and coordination [7]. The neuronal degeneration within the nigrostriatal pathway as well as in minor level in mesolimbic, mesocortical and hypothalamic dopaminergic pathways is accompanied by appearing of protein inclusions termed Lewy bodies (LB) [8, 9]. Deposition of the LB is considered as a main histopathological marker for neuronal degeneration observed in PD [10]. The presence of LB is also observed at lower and phylogenetically older structures of CNS such as olfactory bulbs, ventral tegmental area and spinal cord [11–13]. Currently, it is postulated that the outbreak of degenerative changes in the early, asymptomatic stages of PD, could be located in the neurons composing reticular formation (RF) including the gigantocellular reticular nucleus (Gi) within the brainstem. The progression of the neuropathological cascade ascend to the structures of the midbrain leading to the development of pathological protein [14, 15]. It is still unknown what causes the appearance of these inclusions in certain groups of neurons and what is their role in the initiating of the neurodegenerative process. The neuroanatomical correlates of PD progression has been broadly described by Braak et al. in numerous studies [16]. Authors devised their staging system of PD progression by assessing the regional distribution of α-synuclein (ASN) immunoreactive inclusion bodies in the brainstems. ASN is a small presynaptic protein expressed throughout the CNS, and it is the main component of LB [17]. For many years it has been accepted that the certain form of ASN is the natively unfolded monomer with a molecular weight of approximately ~ 14 kDa with a polypeptide chain composed of 140 amino acid residues [18]. However, in 2011 Bartels et al. showed that helically folded tetramer (~ 58 kDa) is a dominant form of ASN in the CNS [19]. Many experimental and clinical data indicate that an abnormal conformation or excessive accumulation of ASN in the brain can induce neurotoxicity leading to the progressive neurodegeneration observed in PD [20]. A deviation from normal physiological concentrations of ASN can affect the homeostasis of dopaminergic system and induce changes in the synthesis and metabolism of DA [21]. The recent data indicated the possible involvement of transglutaminase 2 (TG2) in several neurodegenerative processes present in PD by catalyzing the formation of protein aggregates [22, 23]. TG2 belongs to the family of transamidating acyltransferases that catalyzes a (Ca^2+^)-dependent protein modifications and may also act as GTPase/ATPase, protein disulfide isomerase and protein kinase [24, 25]. The aim of the present study was to determine the effect of elevated concentrations of recombinant human ASN monomers in the brainstem of male C57BL/10 Tar mice at the initiation and progression of dopaminergic neurons loss in the nigrostriatal pathways defined as the DA and its metabolites concentration and tyrosine hydroxylase (TH) messenger RNA (mRNA) expression in the ST. We examined also the TG2 mRNA expression to assess its potential role in our model. Recombinant human protein ASN monomers was bilaterally administered to the Gi area of the brainstem by stereotactic injection to initiate the neuropathological cascade.

## Materials and Methods

### Animals

**Fig. 1:**
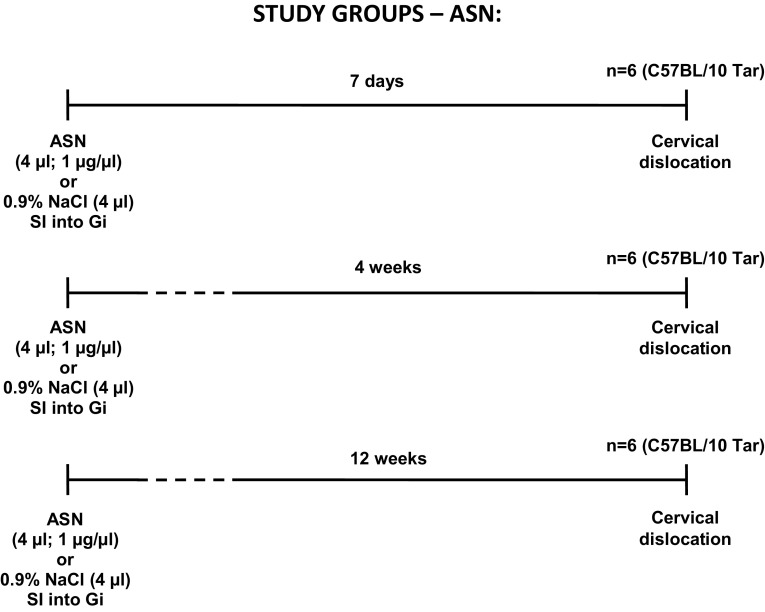
Schematic timelines showing the experiment design and procedures performed on study or control groups. ASN α-synuclein, NaCl saline solution. *SI* stereotactic injection, *Gi* gigantocellular reticular nucleus

Male C57BL/10 Tar mice at the age of 12 months (body weight of 35–38 g) were used in this study. Animals were housed in groups of 3–5/cage in standard laboratory conditions at the controlled temperature (22 ± 5 °C) and 60 ± 5% humidity with a 12 h light/dark cycle (7:00 a.m./7:00 p.m.). All animals were given ad libitum access to food and water. The study groups consist total number of 18 animals (n = 18) with 6 animals (n = 6) in each of three subgroups was distinguished and allocated on the basis of length of the period after performed stereotactic injections (Fig. 1). The control groups of animals (n = 18) were designed in the same manner (Fig. 1). The experimental protocols were approved by the 2nd Local Ethics Committee in Warsaw and were conducted in accordance with European Union (EU) Directive 2010/63/EU on the protection of animals used for scientific purposes and National Research Council (NRC) Committee for the Update of the Guide for the Care and Use of Laboratory Animals (8th edition; National Academies Press 2011). All efforts were made to reduce the number of animals used and to minimize animals suffering.

### Stereotactic Injections

Mice were anesthetized with ketamine (50 mg/ml)/xylazine (20 mg/ml) combination (1:1, v/v) in the dose of 2 ml/kg via intraperitoneal (i.p.) injection. After the induction of anesthesia, mice were placed and their heads were stabilized at three points by two blunt ear bars and anterior bite bar in a stereotactic frame (51900; Stoelting, Wood Dale, IL, USA) with an additional adaptor (51625; Stoelting, Wood Dale, IL, USA). The longitudinal incision was made in the skin overlying the skull exposing both the sagittal and coronal sutures of the skull and then burr-hole was made with a needle just above the infusion site based on the scheduled coordinates. Injection and infusion were performed using a microsyringe pump (UMP2; World Precision Instruments, Sarasota, FL, USA) connected with a programmable controller (Micro 4; World Precision Instruments, Sarasota, FL, USA). A programmable microsyringe pump was used to deliver 4 µl of human recombinant ASN (S7820; Sigma-Aldrich, St. Louis, MO, USA) dissolved in sterile 0.9% saline solution (NaCl) with concentration of 1 µg/µl into Gi at the following stereotactic coordinates—AP (y): − 5.91, ML (x): 0.00 relative to bregma and DV (z): − 5 mm relative to dura (Paxinos G, Franklin KBJ. The Mouse Brain in Stereotaxic Coordinates, 2nd Edition; Academic Press, 2001, San Diego, CA, USA). ASN was administered at estimated 0.5 ml/min rate. After infusion, the scalp was closed with 5–0 monofilament nylon sutures on 16 mm cutting needle of 3/8 circle (DK05PA; Yavo, Poland). Animals from the control groups received identical anesthesia and stereotactic injection with the administration of equal volume of NaCl.

### Tissue Dissection and Preparation

The animals were euthanized by cervical dislocation and decapitated at 1, 4 and 12 weeks after ASN or NaCl injection. The intact brain was rapidly and completely removed from the skull and put on an ice-cold glass plate. Both, right and left ST were microsurgically dissected using a laboratory microscope (SK-292H; Opta-Tech, Warsaw, Poland) with dedicated tools, weighed (XS105 Dual Range; Mettler Toledo, Greifensee, Switzerland) and then immediately placed in dry ice (CO_2_) and stored at − 80 °C until further use. The obtained ST were used for consecutive determination of monoamines and amino acid neurotransmitters assay concentration as well as both, TH and TG2 mRNA expression. All operations were carried out in a highly reproducible manner so as to exclude any significant its impact on the results obtained.

### High-Performance Liquid Chromatography (HPLC) Analysis

#### Assay of Monoamines Concentration

The examined ST concentration of DA and its corresponding metabolites: 3,4-dihydroxyphenylacetic acid (DOPAC), 3-methoxytyramine (3-MT), homovanillic acid (HVA) and 3,4-dihydroxyphenylethanolamine (NA) as well as 5-hydroxytryptamine (5-HT) with it metabolite: 5-hydroxyindolacetic acid (5-HIAA) were determined by HPLC method according to the method described previously by Joniec-Maciejak et al. [26]. The HPLC system consisted of a delivery pump (Mini-Star K-500; Knauer, Berlin Germany), an autosampler automatic injector (LaChrom L-7250; Merck-Hitachi, Darmstadt/Tokyo, Germany/Japan), an electrochemical detector (L-3500A; Merck-Recipe, Darmstadt/Munich, Germany) with a glassy-carbon working electrode. The analysis was performed at a potential + 0.8 V vs. an Ag/AgCl reference electrode. Immediately, before the analysis the striata were homogenized in ice-cold solution containing 0.1 N perchloric acid (HClO_4;_ Sigma-Aldrich, St. Louis, MO, USA) and 0.05 mM ascorbic acid (C_6_H_8_O_6,_ Sigma-Aldrich, St. Louis, MO, USA) and centrifuged (Labofuge 400R; Heraeus Instruments, Hanau, Germany) at 13.000×*g* for 15 min at 4 °C. The supernatant was filtered through a 0.2 µm pore size PTFE membrane syringe filter (6792-1302 Puradisc; Whatman, UK). Monoamines were separated isocratically using EC 250/4 Nucleosil 100-5 C18AB (250 mm length × 4 mm internal diameter, 5 µm particle size, 100 Å) HPLC column (720936.40; Macherey-Nagel, Düren, Germany). The mobile phase comprised 34 mM citric acid (C_6_H_8_O_7_) buffer (Sigma-Aldrich, St. Louis, MO, USA), 32 mM sodium phosphate (NaH_2_PO_4_) buffer (Sigma-Aldrich, St. Louis, MO, USA), 1 mM octane sulfonic acid (C_8_H_18_O_3_S) buffer (Sigma-Aldrich, St. Louis, MO, USA), 54 mM ethylenediaminetetraacetic acid (EDTA) buffer (Sigma-Aldrich, St. Louis, MO, USA) and 12% methanol (Merck, Darmstadt, Germany) in ultrapure water (18 MΩ cm).

The chromatograms were recorded and integrated by use of the computerized data acquisition Clarity software (version 5.0; DataApex, Prague, Czech Republic). The monoamines concentrations were quantified and calculated by comparison with the standard reference solutions (external calibration). All used monoamines standards were purchased from Sigma-Aldrich—St. Louis, MO, USA. The monoamines concentrations in the sample were expressed as pg/mg wet tissue.

#### Assay of Amino Acid Concentration

The examined ST concentration of the amino acids: aspartate (ASP), glutamate (GLU), histidine (HIS), alanine (ALA), taurine (TAU) and γ-aminobutyric acid (GABA) in the samples was performed by HPLC with electrochemical detection according to the method described previously by Joniec-Maciejak et al. [26]. The chromatograph system consisted of an electrochemical detector (EC3000; Recipe, Munich, Germany), an autosampler (Primaide 1210; Hitachi, Tokyo, Japan) and the pump (Primaide 1110, Hitachi, Tokyo, Japan). To obtain agents for derivatisation 22 mg o-phthaldialdehyde (OPA) reagent (Fluka, Buchs, Switzerland) was diluted in 0.5 ml of 1 M sodium sulphite (Na_2_SO_3_), 0.5 ml of methanol, 0.9 ml of 0.1 M sodium tetraborate (Na_2_B_4_O_7_) buffer adjusted to pH 10.4 using 5 M sodium hydroxide (NaOH). The derivatising agent (20 μl) was reacted with amino acid standard (1 ml) or with supernatant samples (1 ml) for 15 min. The separation of amino acids was performed using a Luna 5 µm C18(2) 100 Å (250 mm length × 4.6 mm internal diameter) HPLC reverse phase column (00G-4252-E0; Phenomenex, Torrance, CA, USA). The mobile phase comprised of 45 mM disodium phosphate (Na_2_HPO_4_) buffer (Sigma–Aldrich, St. Louis, MO, USA), 0.2 M citric acid buffer (Sigma–Aldrich, St. Louis, MO, USA) and 0.15 mM EDTA buffer (Sigma–Aldrich., St. Louis, MO, USA) containing 24% methanol (Merck, Darmstadt, Germany). Preparation of the mobile phase and the derivatising agents were based on the method developed by Rowley et al. with minor modifications [27]. Samples were quantified by comparison with standard reference solutions (external calibration) and concentrations calculated with Primaide (version 1.0; Merck, Darmstadt, Germany). All amino acid standards were purchased from Sigma-Aldrich—St. Louis, MO, USA. The concentration of amino acids in the sample was expressed as ng/mg of wet tissue.

### Real-Time Polymerase Chain Reaction (Real-Time PCR)

The striatal mRNA expression levels of TH and TG2 were evaluated using Real-Time PCR. Total RNA isolation was performed using TRI Reagent (Sigma-Aldrich, St. Louis, MO, USA) in accordance with the manufacturer’s instructions. The striatal mRNA expression levels of TH and TG2 were evaluated using Real-Time PCR. Total RNA isolation was performed using TRI Reagent (Sigma-Aldrich, St. Louis, MO, USA) in accordance with the manufacturer’s instructions. The concentration of obtained RNA was measured spectrophotometrically at wavelength of 260 nm (BioPhotometer, Eppendorf AG, Hamburg Germany). The single-strand complementary DNA (cDNA) was synthesized from total RNA using a PrimeScript RT Reagent (Perfect Real Time; Takara Bio, Otsu, Japan) in a SensoQuest Labcycler (SensoQuest GmbH, Göttingen, Germany). The incubation conditions for reverse transcription consisted of 15 min at 37 °C followed by 5 s at 85 °C. Following the reverse transcription reaction, the cDNA products were stored at − 20 °C until further use. Real-Time PCR was performed on a RotorGene Q 5plex HRM System (Qiagen Benelux BV, Velno, The Netherlands). The cDNA was amplified with gene-specific primers designed using the publicly available National Center for Biotechnology Information (NCBI) Primer-BLAST software tool (Table 1). To eliminate sampletosample differences in RNA extraction and conversion to cDNA, we amplified the housekeeping gene glyceraldehyde 3-phosphate dehydrogenase (GAPDH) in each sample. Each PCR mixture contained 1 μl of cDNA along with 10 μl of FastStart Essential DNA Green Master (Roche Molecular Systems, Alameda, CA, USA) and 1.25 μl (10 μM) of each primer in a total reaction volume of 20 μl. The amplification protocol was as follows: initial denaturation at 95 °C for 10 min; 40 cycles at 95 °C for 15 s, 58 °C for 15 s and 72 °C for 15 s. Melting-curve analysis was applied to all reactions in order to ensure the consistency and specificity of the amplified product. All the amplifications were carried out in duplicate. The relative expression of the genes was calculated using Pfaffl method [28].

**Table 1:** Primer sequences used for Real-Time PCR amplification

Target	Real-time PCR primer sequences (5′–3′)		Predicted product size (bp)
TH	F	5′-AACCTACCAGCCGGTGTACT-3′	94
	R	5′-AGAGAATGGGCGCTGGATAC-3′	
TG2	F	5′-TCAGCAAGTGAAGTACGGGC-3′	106
	R	5′-GGCGGAGTTGTAGTTGGTCA-3′	
GAPDH	F	5′-TCTCCCTCACAATTTCCATCCCAG-3′	100
	R	5′-GGGTGCAGCGAACTTTATTGATGG-3′	

*TH* tyrosine hydroxylase, *TG2* transglutaminase 2, *GAPDH* glyceraldehyde 3-phosphate dehydrogenase, *F* forward primer, *R* reverse primer

### Statistical Analysis

Data were analyzed using Statistica 9.0 PL (StatSoft Inc., Tulsa, OK, USA) software package for Windows. To test for differences between the groups Mann–Whitney U test were used. The results were considered statistically significant when p-values were less than 0.05 (p < 0.05). Data are presented as a mean value ± SEM.

## Results

### Effect of ASN Administration on ST Monoamines and Amino Acid Concentration

**Fig. 2:**
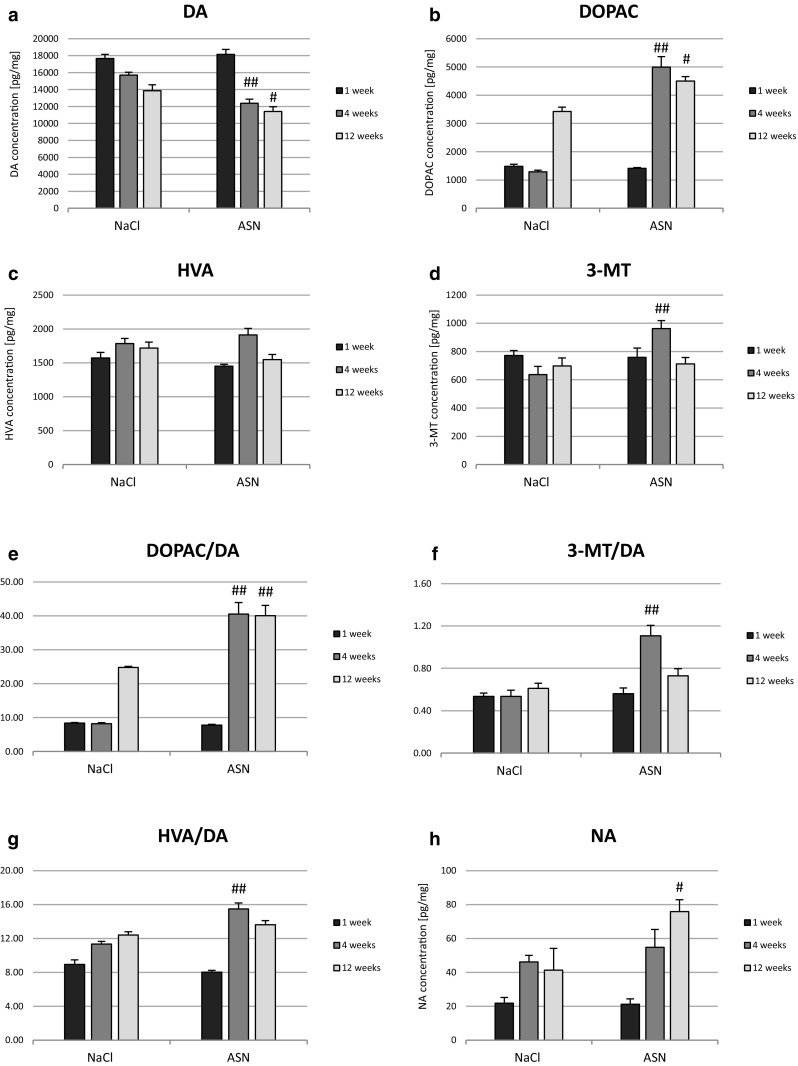

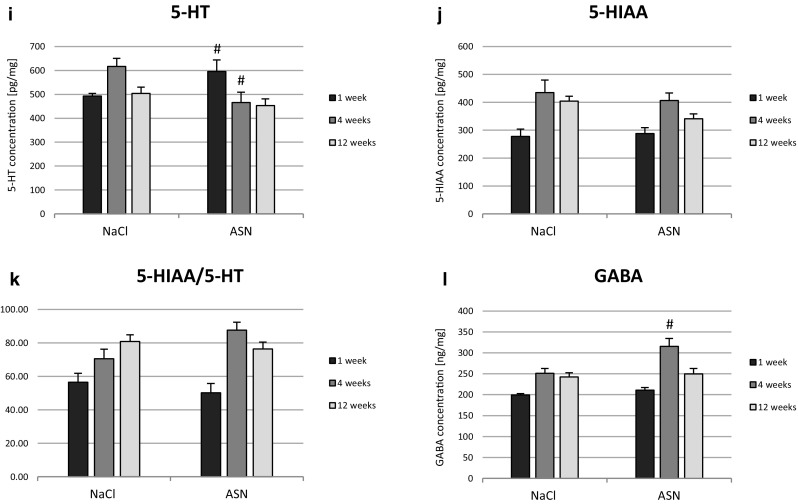
Concentration of DA (**a**), DOPAC (**b**), HVA (**c**), 3-MT (**d**) and DOPAC/DA (**e**), 3-MT/DA (**f**), HVA/DA (**g**) ratio as well as concentration of NA (**h**), 5-HT (**i**), 5-HIAA (**j**), and 5HIAA/5HT (**k**) ratio and GABA (l) concentration in the ST of C57BL/10 Tar mice in 1, 4 and 12 weeks post ASN or NaCl injection into Gi area. Data represent means ± SEM of 6 (n = 6) mice per group. #Differs from NaCl (control) group, ^#^p < 0.05, ^##^p < 0.01

The ASN treatment groups presented reduced DA in ST (Fig. 2a) and increased DOPAC concentration (Fig. 2b) compared with the NaCl groups in 4 and 12 weeks after injection. 3-MT concentration was increased 4 weeks after ASN administration (Fig. 2d). The level of HVA persisted unchanged (Fig. 2c). The DA turnover rate was estimated by the ratio of the DOPAC to DA, the ratio of 3-MT to DA and the ratio of HVA to DA. ASN administration caused an increase in dopamine turnover (DOPAC/DA) in 4 and 12 weeks (Fig. 2e) and both, 3-MT/DA and HVA/DA ratios in 4 weeks compared to NaCl groups (Fig. 2f-g). ASN injection increased 5HT concentration at 1 week but on the contrary reduced at 4 weeks (Fig. 2i) whereas the level of 5-HIAA, a serotonin metabolite, remained unchanged (Fig. 2j). The ASN treatment also had no effect on the ratio of 5-HIAA/5-HT as an index of 5HT turnover (Fig. 2k). ASN injection led to the increase of GABA (p < 0.05) (Fig. 2l) and HIS concentration at time point of 4 weeks. ASN had no influence on the TAU, ALA, ASP and GLU levels in ST compared to the control groups.

### Effect of ASN Administration on ST mRNA Expression for TH and TG2

**Fig. 3:**
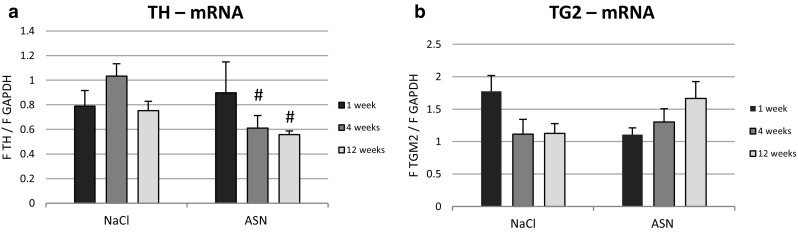
Changes in the expression of mRNA for TH (**a**) and TG2 (**b**) normalized to GAPDH in the ST of C57BL/10 Tar mice 1, 4 and 12 weeks post ASN or NaCl injection into Gi area. The data are presented as the mean ± SEM of 6 (n = 6) mice per group. #Differs from NaCl (control) group, ^#^p < 0.05

ASN treatment reduced TH mRNA expression in ST compared to the control groups in 4 and 12 weeks after ASN or NaCl injection into Gi (Fig. 3a). The TG2 mRNA expression in ST remained unchanged post ASN administration into Gi (Fig. 3b).

## Discussion

The pathogenesis of PD is characterized by a slow but progressive and long-lasting process of dopaminergic neuronal degeneration [29]. This multistep cytopathological phenomenon is occurring mainly within non-myelinated fibers and specific structures in CNS successive affecting its higher systems and neuroanatomical regions [30, 31]. The initial neuropathological changes are observed in medulla oblongata, ventral tegmental area and olfactory bulbs [32]. According to the staging model proposed by Braak et al. this first asymptomatic phase (stage 1 and 2) is characterized by lack of the clinically detectable symptoms [33]. In this case clinical presentation occurs at the time of neurodegeneration occurring within SN and other nuclei situated in midbrain and forebrain (stage 3 and 4). Advanced and severe lesions are characterized by degeneration within neocortex (stage 5 and 6). Despite numerous pre- and clinical studies, we do not yet know the phenomena in regard of initiation of neuropathological changes, but many potential factors are currently postulated [34]. Insights afforded by detailed research indicate the significance of the excessive deposition and toxicity of ASN within CNS [35]. Nevertheless, ASN is a constitutive protein widely expressed by several neuronal populations serving in the regulation of neurotransmission and mobilization of synaptic vesicles, including DA metabolism and turnover [36]. It was observed that generated ASN^−/−^ hybrid 129SV/j × C57BL/6 F2 mice exhibited a reduction of DA levels in ST associated with attenuation of DA-dependent locomotor response to amphetamine (C_9_H_13_N) [37]. As it was mentioned, the predominant physiological type of ASN is related to its native folded tetrameric form which in different conditions could undergo structural modification leading to its disassemblyand generation of unstable monomeric form [38, 39]. These liberated monomeric conformers of ASN present susceptibility to misfolding and pathological aggregation forming oligomers which are mainly responsible for neuronal toxicity and increased presence of abnormal spherical structures referred to as LB [40]. The LB constitute cytoplasmic inclusions which are characteristic for patients with PD and other synucleinopathies [41]. In pathological terms the unfavorable dyshomeostasis of tetrameric multimers and an absolute increase of ASN monomers proportion could influence DA biosynthesis regulation and its mediated neurotransmission [42, 43]. According to the current understanding the originof the neuropathological changes in early phase of the disease is situated within medulla oblongata nuclei including the Gi and its interconnections [44]. In accordance with these findings the purpose of our study was to evaluate effects of direct injection of ASN monomers into Gi area in which the occurrence of primary changes in the course of PD was postulated. To our knowledge, this is the first study to analyze that phenomenon after direct intracerebral administration of human recombinant ASN monomers into Gi area in mice. After ASN administration we have observed changes regarding DA concentration in ST which were visible 4 weeks after injection. Then a significantly lower concentration of DA in ST relative to NaCl group was demonstrated. The same changes were observed 12 weeks after ASN administration. At these time points, we also observed decreasing TH mRNA expression in ST which was involved in the production of DA. Previous studies have shown that overexpression of ASN has been found to inhibit expression and activity of TH [45]. In 4 and 12 weeks after ASN administration we also observed a tendency for increased TG2 mRNA expression which was not significant. TG2 is the enzyme which catalyzes the deamination and transacylation of proteins [46]. The control of protein oligomerization by TG2 is a cause for the formation of insoluble macromolecular aggregates [47]. ASN inclusions are present in degenerating neurons in Parkinson’s disease [48]. Overexpression of TG2 has been observed in apoptotic cells [49]. The study of Andringa et al. demonstrated that many of the ASN monomers in the course of PD are crosslinked by TG2 and thus the function of SN may be impaired [50]. This modification appears to be an early step in PD pathogenesis, preceding the aggregation of ASN in Lewy bodies [51]. These findings correspond with a theory proposed by Braak et al. that the first neuropathological changes, including deposits of ASN, are observable within brainstem and then with time affecting higher neuroanatomical regions [32, 33]. At the same time points (4 and 12 weeks) after injection we found a significantly higher concentration of the DOPAC. In 4 weeks after administration of ASN we also found a higher concentration of 3-MT. These data indicate that the increased concentration of ASN in Gi area is associated with the increase of DA metabolism in ST. This observation is also confirmed by the significant increase in DA turnover in ST determined as ratios: DOPAC/DA, 3-MT/DA and HVA/DA. On this basis we can assume that this is most likely an effect of increased activity of neurochemical DA metabolizing enzymes such as monoamine oxidase B (MAO-B) and catechol-O-methyltransferase (COMT) [52]. In addition, DA metabolism is linked to the concomitant production of DOPAC and hydrogen peroxide (H_2_O_2_) which may be responsible for neuronal damage [53]. Directly administrated ASN initiates neurotoxic changes, reflecting the early stage of PD. However, we are of an opinion that the single injection of ASN may not be enough to intensify these changes. We suppose that the ASN potentially degraded because it was unable to demonstrate its presence by immunohistochemical (IHC) staging. It corresponds with the results demonstrated by Masuda-Suzukake et al. which indicate that ASN degrades after 5–7 days after administration [54]. In accordance with data considering serotonergic transmission, we have shown that ASN injection into Gi results in a 5-HT increase in ST at 1 week after but the decrease at 4 weeks after infusion. On the contrary, there was no influence of ASN administration on ST concentration of 5-HIAA and there was no change in 5-HT metabolism calculated as 5-HIAA/5-HT ratio. This is consistent with a slow and non-linear dysfunction of serotonergic transmission due to its associated neuron loss observed in positron emission tomography (PET) examination, indirectly confirming the specificity of our model [55]. As PD is associated with the imbalance of the entire neurotransmitter system, we have performed an assay to asses amino acid concentration [56, 57]. Typical changes such as increased concentration of HIS and GABA were observed in 4 weeks after ASN administration. Our research was based on a direct stereotactic injection of ASN monomers into the Gi area, where primary changes in the course of PD are postulated. This is a new mouse model of PD and should be useful for elucidating progression mechanisms and evaluating disease-modifying therapy.
